# Nature imagery on cigarette vending machines in Ireland: A lacuna in tobacco control

**DOI:** 10.18332/tpc/113646

**Published:** 2019-11-27

**Authors:** Frank Houghton

**Affiliations:** 1 Limerick Institute of Technology, Limerick, Ireland

**Keywords:** tobacco control, cigarette vending machines, nature imagery, children

## Abstract

Legislation in Ireland now requires standardised packaging for cigarette packets as well as the EU mandated combined graphic and text anti-smoking warnings. However, although overt tobacco advertising has also been banned for many years in Ireland, a lacuna currently exists in relation to cigarette vending machines. An examination of industry practice has identified the use of bucolic and coastal scenes on the outside of vending machines. This is problematic for three reasons. First, they are reminiscent of former cigarette advertisements and packaging. Second, such artwork serves to minimise the environmental damage caused by the tobacco industry and their products. Third, the use of landscape imagery undermines the Irish Government’s strategy of denormalising smoking.

## COMMENTARY

Recent years have witnessed dramatic advances in tobacco control in many countries including Ireland. Restrictions on tobacco advertising in Ireland are longstanding, and include bans in both the media and at the point-of-sale. Ireland has also recently introduced mandatory plain packaging for cigarette packs (featuring Pantone 448 C, often described as the world’s ugliest colour)^1^. This follows on from prior EU legislation that had already introduced combined graphic and text anti-smoking warnings, some years ago. Thus, cigarette packets may now be referred to as dissuasive^2^.

However, having made the cigarette packets aversive and having outlawed not only in-store advertising, but also the open display of cigarette packets, the question of the appearance of cigarette vending machines in Ireland remains. Current Irish legislation around such machines requires the packets themselves to be effectively hidden, and clearly prohibits tobacco advertising. However, unlike some other countries such as New Zealand^3^, beyond these basic stipulations in Ireland there is little or no guidance. In contrast to its Information for Retailers document, for example, the Ministry of Health in New Zealand suggests^3^:

‘Consider restocking when your premises are closed or during quiet times. If you restock during busy times, there is an increased risk that tobacco products will be left in the view of the public…’

‘Don’t leave the restocking process to serve customers or do other tasks unless you remove the tobacco products from their sight and close the cabinets’.

It should be noted that the vast majority of European countries have already banned cigarette vending machines, often because of evidence demonstrating the importance of this route in youth access to tobacco products^4^. Increasingly sophisticated measures designed to restrict access to tobacco products from vending machines to adults only, have largely proven ineffective^5-9^.

The cigarette vending machine industry in Ireland is represented by the Irish Cigarette Machine Operators Association (ICMOA) and has over 60 members. This group has fought vociferously for the right to continue operating their trade in Ireland, and undoubtedly wish to make their machines appear aesthetically pleasing to ensure widespread acceptance of their machines. However, the ‘grey area’ in legislation has facilitated the widespread adoption of imagery on cigarette vending machines in Ireland that is problematic. It should be noted that Big Tobacco has an established track record of evading and subverting advertising controls. Henriksen^10^ has outlined many of these:

‘adopting weak voluntary advertising codes to avoid stronger measures, hiring consultants to discredit the evidence base for restrictions, using third-party companies to lobby against marketing restrictions, delaying or weakening their implementation with protracted litigation, and publicising imaginary barriers to enforcement…’.

**Figure 1:**
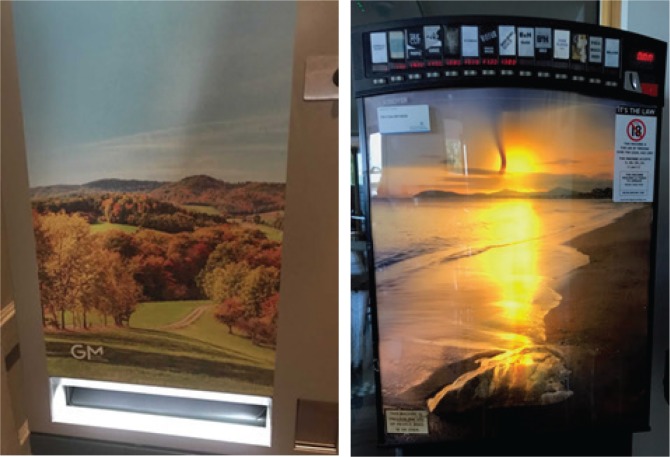
Examples of images routinely displayed on cigarette vending machines in Ireland: Rustic and coastal scenes

Big Tobacco has also been guilty of blatant misrepresentation in their advertising in efforts to elude controls. Three examples, in particular, evidence these practices. In 1988 Philip Morris launched in Singapore a new brand, Alpine cigarettes. In order to circumvent advertising restrictions on tobacco products there, the launch of the new cigarette brand was preceded by an advertising campaign by Philip Morris promoting the Alpine wine cooler^11^. A second example may be seen in the field of athletics. Examination of the 1983 Bislett Games in Norway reveals advertising for Silk Cut cigarettes. However, in order to bypass recently introduced legislation, the memorable style and colouring of the words Silk Cut are retained but the words Masterclass Holidays was added afterwards in a weak attempt at deception^12-13^. Perhaps the best known example of Big Tobacco’s blatant contempt for advertising restrictions may be seen in relation to the Scuderia Ferrari Formula 1 team. Marlboro cigarettes had a long history of sponsoring the team when advertising restrictions from the 2005 European Union Tobacco Advertising Directive outlawed such advertising. Marlboro cigarettes responded by adopting what has been termed an evolving ‘barcode’ design, which they claimed was simply part of the livery of the racing car. This ‘barcode’ design retained the symbolic red, white and black colouring of the Marlboro brand and was revealed as an example of ‘alibi marketing’^14^. Pastoral woodland scenes such as those in Figure 1 often appear on Irish cigarette vending machines. Such depictions are problematic for three reasons.

First, they are remarkably reminiscent of countryside images that featured extensively in some early UK cigarette package art. Mullan^15^ notes that: *‘Cigarettes have long been associated with the Great Outdoors… Some of the earliest images associated with cigarette packs concerned the enjoyment of the open air, the evocation of the sweeping landscape’.* The use of such bucolic imagery has associations with being healthy and active and enjoying ‘nature’ filled environments.

The incorporation of such therapeutic landscape imagery on cigarette packets was, for example, particularly notable on Beechwood and Lloyd’s Big Tree brands, which claimed to *‘offer to the smoker the lure of the leafy forest glade… Both present a rustic seclusion with healthy living and dappled light, a celebration of the… countryside that appeals to both the romantic and the patriot’.* Plant imagery was also standard on well-known packs historically such as (Wills) Wild Woodbine^15^.

Figure 1 also presents an alternate therapeutic landscape that is also often portrayed on cigarette vending machines in Ireland. This seashore scene is also very similar to that previously used on numerous cigarette packets such California Lights by Philip Morris, Pinkerton Tobacco Company’s Sunshine Cigarettes, and the Cia Industrial del Centro’s Faros brand from Mexico^16^.

Second, the use of such nature imagery is highly problematic as it is essentially a form of ‘greenwashing’ a highly toxic product that causes extensive environmental damage^17^. The use of appealing pastoral and coastal scenes serves to underplay the significant negative impact of tobacco from the earliest stages of production, to disposal in the form of cigarette butts^18-20^. A cradle-to-grave approach to tobacco and cigarette production and disposal is required. The UN has explored in-depth the significant negative environmental impact of the global tobacco industry^21^.

Third, nature imagery on cigarette vending machines is also an issue as the incorporation of such pastoral and coastal scenes serves to normalize tobacco products. Nature imagery is a standard feature on numerous commercially available products and the use of this imagery on vending machines serves to depict cigarettes as ‘just another product’. The Irish government supports the denormalization of smoking as part of its strategy to combat smoking. As part of its denormalization strategy the Irish Government has set a target^22^ of becoming officially smoke-free (i.e. having an adult smoking prevalence of <5%) by 2025. However, even this modest target^23^ is likely to be missed by decades^22,24^.

Concerns over cigarette vending machines in Ireland are by no means new. The Irish Government previously aimed to ban the sale of cigarettes from vending machines in both 2014 and 2016, a move that was never implemented, presumably as a result of vocal opposition from industry representatives^25-27^.

## CONCLUSION

In relation to denormalising smoking, the best option is clearly for Ireland to follow almost all of its European neighbours and finally impose a complete ban on cigarette vending machines. This option has once again been suggested^28^. Alternatively, the outer casing of cigarette machines could be legally mandated to require the display of the EU’s combined anti-smoking graphics, or a stop-smoking quitline number. A final option could be for the vending machines to simply be required to feature a monotone dissuasive colour, such as Pantone 448 C mentioned above^1^.
